# The Newport Meeting of the American Medical Association

**Published:** 1889-07

**Authors:** 


### *THE NEWPORT MEETING OF THE AMERICAN MEDICAL ASSOCIATION.*

SELDOM has it fallen to the lot of this time-honored medical body to cast its lines in a more pleasant place than it did at its fortieth annual meeting that has just been held. True, it has had a larger attendance in times gone by, and it has met in many larger cities ; but it never found warmer hearts to greet it, and it has seldom done better work, or had greater harmony in its deliberations. In the election of Dr. E. M. Moore, of Rochester, to the presidency, it has performed a graceful act to a man who is the peer of any American physician.

In the selection of Nashville as its next place of meeting, we feel sure it has acted wisely. Other cities made strong claims to be its host in 1890, but none can treat it more generously, according to its ability, than this lovely city of the mid-South. There need be no fear as to accommodations for all who will attend—we are assured they will be ample.

The Rhode Island Medical Society deserves all praise for its generous acts of hospitality, and the clam-bake which it provided at Rocky Point on Friday, June 28th, will ever be remembered by the 1,000 guests present as a royal ending to the Newport meeting.

Western New York may well feel honored that the Association came to this region for its President, and that it chose Dr. Moore; for no man has a stronger hold upon the affections of the profession here. It was in Buffalo that for thirty years he taught surgery in some of its branches, and it does no one injustice to say that no teacher has ever excelled him in the exposition of the science and art of surgery. His philosophic mind and well-trained touch peculiarly equip him to teach and practice that art which Sir Astley Cooper said required "an eagle's eye, a lion's heart, and a lady's hand," to deal with successfully. Dr. Moore, without exaggeration, may be said to be an ideal surgeon, at least as nearly so as frail humanity can ever be, and we but voice the sentiment of the profession in this region, when we extend to him cordial congratulations upon entry into this high office,—albeit, an office that thus filled reflects quite as much honor upon itself as upon him.

Other officers chosen from this region were as follows:—Section of *Laryngology and Otology*: Dr. John O. Roe, of Rochester, Chairman, and Dr. Frank H. Potter, of Buffalo, Secretary. Section of *Obstetrics and Diseases of Women*: Dr. W. W. Potter, of Buffalo, Chairman, and Dr. Joseph Hoffman, of Philadelphia, Secretary.

